# The BAG Homology Domain of Snl1 Cures Yeast Prion [URE3] Through Regulation of Hsp70 Chaperones

**DOI:** 10.1534/g3.113.009993

**Published:** 2014-03-13

**Authors:** Navinder Kumar, Deepika Gaur, Daniel C. Masison, Deepak Sharma

**Affiliations:** * Council of Scientific and Industrial Research-Institute of Microbial Technology, Chandigarh, India; † Laboratory of Biochemistry and Genetics, National Institutes of Diabetes and Digestive and Kidney Diseases, Bethesda, Maryland

**Keywords:** Hsp70, nucleotide exchange factor, yeast prion, Snl1, BAG domain

## Abstract

The BAG family of proteins is evolutionarily conserved from yeast to humans and plants. In animals and plants, the BAG family possesses multiple members with overlapping and distinct functions that regulate many cellular processes, such as signaling, protein degradation, and stress response. The only BAG domain protein in *Saccharomyces cerevisiae* is Snl1, which is anchored to the endoplasmic reticulum through an amino-terminal transmembrane region. Snl1 is the only known membrane-associated nucleotide exchange factor for 70-kilodalton heat shock protein (Hsp70), and thus its role in regulating cytosolic Hsp70 functions is not clear. Here, we examine whether Snl1 regulates Hsp70 activity in the propagation of stable prion-like protein aggregates. We show that unlike other nucleotide exchange factors, Snl1 is not required for propagation of yeast prions [URE3] and [*PSI^+^*]. Overexpressing Snl1 derivative consisting of only the BAG domain (Snl1-S) cures [URE3]; however, elevated levels of the entire cytosolic domain of Snl1 (Snl1-M), which has nine additional amino-terminal residues, has no effect. Substituting the three lysine residues in this region of Snl1-M with alanine restores ability to cure [URE3]. [*PSI^+^*] is unaffected by overproduction of either Snl1-S or Snl1-M. The Snl1-S mutant engineered with weaker affinity to Hsp70 does not cure [URE3], indicating that curing of [URE3] by Snl1-S requires Hsp70. Our data suggest that Snl1 anchoring to endoplasmic reticulum or nuclear membrane restricts its ability to modulate cytosolic activities of Hsp70 proteins. Furthermore, the short amino-terminal extension of the BAG domain profoundly affects its function.

BAG domain family proteins have a major role in modulating 70-kilodalton heat shock protein (Hsp70) activities and are characterized by the presence of a BAG domain that mediates its interaction with Hsp70 proteins. BAG domain proteins are conserved across eukaryotic species from yeast to humans and also are found in plants. The human BAG family consists of six members (Bag1-6) which differ primarily by the presence of amino acid extensions at the amino terminus of a C-terminal BAG domain (Kabbage and Dickman 2008). This variation specifies the function as well as cellular localization of BAG domain proteins (Alberti *et al.* 2003). Bag-1 (Bcl-2-associated athanogene), one of the first identified BAG proteins, is involved in cellular apoptosis, DNA binding, and transcription. In the presence of Hsp40, BAG domain proteins stimulate Hsp70 ATPase activity as well as substrate release, indicating the nucleotide exchange ability of these proteins. However, in contrast to other nucleotide exchange factors (NEFs), the BAG domain−containing proteins are known to both positively and negatively regulate Hsp70 activity (Hohfeld and Jentsch 1997; Gassler *et al.* 2001). Additional interacting partners of BAG proteins include signaling molecules Siah, steroid hormone receptors (Knapp *et al.* 2012), retinoic acid receptors (Liu *et al.* 1998), ribosome (Verghese and Morano 2012), and Hsp70 substrates. Some of the BAG partners such as protein kinase RAF-1 compete with Hsp70 for Bag-1 interaction, indicating an overlap with Hsp70 interacting site (Song *et al.* 2001), which provides another mode of regulation of Hsp70 activity. BAG domain proteins are thus multifunctional proteins that regulate various cellular processes, such as cellular proliferation, protein degradation, nucleocytoplasmic export, and stress response.

Snl1 is the yeast homolog of mammalian Bag-1 protein, which consists of a transmembrane domain (residues 1−39) and a cytosolic domain (residues 40−159) (Ho *et al.* 1998). The entire cytosolic region of Snl1 consists of a Bag-1 homology domain (residues 49−159) and a nine-amino acid N-terminal extension. As expected from the presence of its transmembrane domain, Snl1 is localized on endoplasmic and nuclear membrane. Similar to mammalian BAG domains, the BAG domain of Snl1 interacts with cytosolic Hsp70 and acts as its NEF (Sondermann *et al.* 2002). Snl1 was originally identified as the high copy suppressor of lethal Nup116-C phenotype (Ho *et al.* 1998). Its ability to suppress Nup116-C phenotype is dependent on its interaction with cytosolic Hsp70s (Sondermann *et al.* 2002). Snl1 is also known to interact with ribosomal proteins and thus plays an important role in translation (Verghese and Morano 2012).

Yeast prions, which propagate as self-perpetuating infectious amyloid aggregates, have been extensively studied as models of mammalian prions and other amyloid diseases. [*PSI^+^*], [URE3], [*PIN^+^*], and [*SWI^+^*] are well-characterized yeast prions of native proteins Sup35, Ure2, Rnq1, and Swi1, respectively (Wickner 1994; Sondheimer and Lindquist 2000; Patel and Liebman 2007; Du *et al.* 2008). The presence of asparagine- and glutamine-rich domains is a common feature among most yeast proteins that form prions, although a recently identified yeast prion, MOD5, lacks such a domain (Suzuki *et al.* 2012; Suzuki and Tanaka 2013). For stable prion propagation in dividing yeast cells, the infectious material needs to efficiently grow and replicate. Proteins belonging to the heat shock family contribute to prion replication and thus alteration in level or activity of these protein chaperones influence yeast prion propagation (Chernoff *et al.* 1995; Moriyama *et al.* 2000; Hung and Masison 2006; Newnam *et al.* 2011; Reidy and Masison 2011).

[*PSI^+^*] and [URE3] are the most widely studied yeast prions. The conversion of Sup35 and Ure2 into [*PSI^+^*] and [URE3], respectively, results in partial loss of their native cellular function. Sup35 is a translation termination factor, and Ure2 is involved in nitrogen metabolism. By binding to transcription factor Gln3, Ure2 suppresses expression of genes required for uptake of poor nitrogen sources when a good nitrogen source is present. Amyloids formed from purified Ure2 and Sup35 prion domains possess in-register parallel beta sheet architecture (Tanaka *et al.* 2004; Brachmann *et al.* 2005; Shewmaker *et al.* 2006; Baxa *et al.* 2007; Ngo *et al.* 2011).

Hsp70 belongs to a family of heat shock proteins that play important roles in several cellular processes, including maintaining protein homeostasis, protein translocation (Shi and Thomas 1992; Su and Li 2010) and cellular signaling. Hsp70’s role is primarily dependent upon its co-chaperones, which not only enhance activity but also confer functional specificity. Hsp70 consists of a nucleotide binding domain (NBD), substrate binding domain, and a C-terminal domain. ATP binding at the NBD opens up the substrate binding pocket present in substrate binding domain. Binding of substrate causes conformational changes in the NBD that allow binding of co-chaperones such as Hsp40 family proteins. Hsp40 proteins stimulate ATP hydrolysis, leading to closing of a lid over the substrate binding domain and substrate gets trapped (Cyr *et al.* 1992). NEFs subsequently interact at the NBD of Hsp70 and promote the release of ADP followed by ATP binding to begin a new cycle (Liberek *et al.* 1991). The rebinding and release of partially folded substrates help them to refold and if folding fails, the substrate is generally targeted for degradation.

*Saccharomyces cerevisiae* contains four Ssa family Hsp70s (Ssa1-4), among which Ssa1 and Ssa2 are constitutively expressed whereas, Ssa3 and Ssa4 are stress inducible. As different Hsp70 members perform both overlapping as well as distinct functions, the presence of multiple members not only ensures an increase in overall abundance during stress but also enables them to perform a variety of essential functions required for cellular fitness. Though highly homologous, different Ssa members affect yeast prions differently (Jung *et al.* 2000; Schwimmer and Masison 2002; Sharma and Masison 2008, 2011; Sharma *et al.* 2009a). When expressed as the sole source of Ssa Hsp70, Ssa2 supports stable [URE3] propagation, but Ssa1 antagonizes [URE3]. Although the underlying mechanism of distinct Ssa Hsp70 functions is still unclear, it is clear that the ATPase domain of Hsp70 governs this distinction (Sharma and Masison 2011).

Although the Sse1 and Fes1 NEFs are cytosolic, Snl1 is anchored to the *endoplasmic reticulum (*ER) and nuclear membrane with its BAG domain facing the cytosol. It is not clear whether Snl1 participates in modulating Hsp70 activities required for its cytosolic functions. Here, we examined the role of Snl1 on propagation of yeast prions [URE3] and [*PSI^+^*], which are known to be remodelled by Hsp70 action. We show that Snl1 is not essential for propagation of [URE3] or [*PSI^+^*]. We found that the BAG domain alone of Snl1 efficiently cured [URE3], but the entire cytosolic domain failed to do so. We also show that curing is specific for [URE3] and not [*PSI^+^*], and requires Snl1 interaction with Hsp70.

## Materials and Methods

### Strains and plasmids

Table 1 and Table 2 describe the strains and plasmids, respectively, used in this study. Strain SY187 is *Mata*, *kar1-1*, *SUQ5*, *P_DAL5_*::*ADE2*, *his3Δ202*, *leu2Δ1*, *trp1Δ63*, *ura3-52*. Strain A1 or A2 is *ssa1*::*KanMX*, *ssa2*::*HIS3*, *ssa3*::*TRP1*, *ssa4*::*ura3-2f* and carries single-copy, *LEU2*-based plasmid with *SSA1* or *SSA2*, respectively, under control of the *SSA2* promoter. The gene encoding Snl1-L was synthesized commercially (Integrated DNA Technologies) and further subcloned into plasmid pRS316 under galactose-inducible *GAL1* promoter with the restriction enzymes *Bam*HI and *Xho*I to generate plasmid pRS316-GAL-SNL1-L. The plasmid pRS316-GAL-SNL1-M or pRS316-GAL-SNL1-S was constructed by replacing *SNL1-L* with *SNL1-M* or *SNL1-S*, respectively, using *Bam*HI and *Xho*I. Plasmid pRS426-GPD-SNL1-S or pRS413-TEF-SNL1-S was constructed by subcloning *SNL1-S* from pRS316-GAL-SNL1-S into pRS426-GPD and pRS413-TEF, respectively, using enzymes *Bam*HI and *Xho*I (Mumberg *et al.* 1995). For protein purification, *SNL1-S* was subcloned into pPROEX-HTb-SSA2 (Sharma and Masison 2011) by replacing gene encoding Ssa2 with Snl1-S using *Bam*HI and *Xho*I to generate pPROEX-HTb-SNL1-S. The plasmid encodes from the 5′ to 3′ direction, has a Hexa-His-tag (His_6_-tag), and has a TEV protease recognition site at N-terminus of Snl1. Similarly, other Snl1 derivatives were subcloned into with pPROEX-HTb plasmid for expression and purification in *Escherichia coli*. For immunoblot analysis of Snl1 and its derivatives from the yeast lysate, a short tract of Hexa-His-tag was added to the C-terminus of the protein using standard DNA recombinant technology.

**Table 1:** Strains used in the present study

Strain	Genotype	Reference
SY187	*MATa*, *kar 1-1*, *SUQ5*, *P_DAL5_*::*ADE2*, *his3Δ202*, *leu2Δ1*, *trp1Δ63*, *ura3-52*, [URE3]	Sharma and Masison 2011
SY206	*MATa*, *kar 1-1*, *SUQ5*, *P_DAL5_*::*ADE2*, *his3Δ202*, *leu2Δ1*, *trp1Δ63*, *ura3-52*, *Snl1*::*KanMX*, [URE3]	This study
SY135	*MATa*, *P_DAL5_*::*ADE2*, *ssa1*::*Kan*, *ssa2*::*HIS3*, *ssa3*::*TRP1*, *ssa4*::*URA3-2f/pRS315-SSA1*, [URE3]	Sharma and Masison 2011
SY136	*MATa*, *P_DAL5_*::*ADE2*, *ssa1*::*Kan*, *ssa2*::*HIS3*, *ssa3*::*TRP1*, *ssa4*::*URA3-2f/pRS315-SSA2*, [URE3]	Sharma and Masison 2011
SY358	*MATa*, *kar 1-1*, *SUQ5,ade2-1*, *his3Δ202*, *leu2Δ1*, *trp1Δ63*, *ura3-52*, *snl1*::*KanMX*, [*PSI^+^*]	This study
SY400	*MATa*, *kar 1-1*, *SUQ5*, *ade2-1*, *his3Δ202*, *leu2Δ1*, *trp1Δ63*, *ura3-52*, [*PSI^+^*]	Jung *et al.* 2001
SWY027	*MATα*, *nup116-5*::*HIS3*, *ade2-1*, *ura3-1*, *his3-11,15*, *trp1-1*, *leu2-3,112*, *can1-100*	Ho *et al.* 1998

**Table 2:** Plasmids used in the present study

Plasmid	Marker	Reference
pRS316-GAL-SNL1-L	URA3	This study
pRS316-GAL-SNL1-M	URA3	This study
pRS316-GAL-SNL1-S	URA3	This study
pRS316-GAL-SNL1-S(E112AR141)	URA3	This study
pRS316-GAL-SNL1-L-His_6_	URA3	This study
pRS316-GAL-SNL1-M-His_6_	URA3	This study
pRS316-GAL-SNL1-S-His_6_	URA3	This study
pRS316-P_SNL_-SNL1-S	URA3	This study
pRS316-GAL-SNL1-S(L114V)	URA3	This study
pRS316-GAL-SNL1-S(L114F)	URA3	This study
pRS316-GAL-SNL1-S(S89N)	URA3	This study
pRS316-GAL-SNL1-S(L114V)-His_6_	URA3	This study
pRS316-GAL-SNL1-S(L114F)-His_6_	URA3	This study
pRS316-GAL-SNL1-S(S89N)-His_6_	URA3	This study
pRS316-GAL-SNL1-M(KKK)	URA3	This study
pRS316-GAL-SNL1-L(KKK)	URA3	This study
pRS426-GPD-SNL1-S	URA3	This study
pRS413-TEF-SNL1-S	HIS3	This study
pRS413-TEF-SNL1-S-His_6_	HIS3	This study
pRS426-GAL-SNL1-L(L114V)	URA3	This study
pRS426-GAL-SNL1-L(L114F)	URA3	This study
pRS426-GAL-SNL1-L(S89N)	URA3	This study
pSW171	TRP1	Ho *et al.* 1998
pSW534	URA3	Ho *et al.* 1998
pPROEX-HTb-SSA2	Ampicillin	Sharma and Masison 2011
pPROEX-HTb-SNL1-S	Ampicillin	This study
pPROEX-HTb-SNL1-M	Ampicillin	This study
pPROEX-HTb-SNL1-M(KKK)	Ampicillin	This study
pPROEX-HTb-SNL1-S(E112AR141A)	Ampicillin	This study
pPROEX-HTb-SNL1-S(L114V)	Ampicillin	This study
pPROEX-HTb-SNL1-S(L114F)	Ampicillin	This study
pPROEX-HTb-SNL1-S(S89N)	Ampicillin	This study

### Media and growth conditions

The strains were normally grown at 30° unless otherwise stated. Plates were poured with 1/2YPD media containing 0.5% yeast extract, 2% peptone, 2% dextrose, and 2% agar. Liquid-rich media yeast extract-peptone-dextrose medium + adenine (YPAD) is similar without agar and contains 1% yeast extract with 200 mg/L adenine. Synthetic-dextrose minimal (SD) media contain yeast nitrogen base (6.7 g/L) and 2% dextrose. SGal media is same as SD except 2% raffinose and 2% galactose are added in place of dextrose. The amino acids were supplemented as needed. To monitor color phenotype adenine was added in limiting amount (9 mg/L) onto SD or SGal plates.

### Monitoring [*PSI^+^*] and [URE3]

The native Ure2 represses *DAL5* promoter in standard ammonium containing media. Thus, when Ure2 is present in native form, *ADE2* gene present under *DAL5* promoter is not transcribed. In the absence of Ade2 synthesis, cells are unable to grow in medium lacking adenine and remain red if grown in limiting adenine medium. The conversion of ure2 into its prion form [URE3] relieves repression of *DAL5* promoter, and thus [URE3] cells are ADE^+^ and show normal white color phenotype.

Sup35 is involved in translational termination. Our *ade2-1* yeast strain expressing soluble Sup35 does not produce Ade2. Thus, [*psi^−^*] cells require exogenous adenine to grow and remain red on medium containing limiting adenine. The conversion of Sup35 into [*PSI^+^*] reduces translation termination efficiency of Sup35, which causes suppression of *ade2-1* nonsense allele. Thus [*PSI^+^*] cells remains white and can grow in the absence of externally added adenine.

### Random mutagenesis of Snl1-S

*SNL1-S* was randomly mutagenized with the Forsburg hydroxylamine mutagenesis−based method (Rose and Fink 1987). Approximately 10 µg of plasmid encoding wt Snl1-S was incubated with 500 µL of mutagenesis buffer (0.35 g hydroxylamine hydrochloride, 450 µL of 5 M NaOH, 4.55 mL of ice-cold sterile MQ water, pH 6.7) for 20 to 48 hr at 37°. Mutant library thus obtained was further purified using QIAGEN PCR clean up kit and used for further transformation.

### Nup116-C growth assay

Plasmids pSW171 encoding Nup116−C and pSW534 encoding wt Snl1-L under galactose-inducible promoter and yeast strain SWY027 (*MATα nup116-5*::*HIS3*, *ade2-1*, *ura3-1*, *his3-11*, *15*, *trp1-1*, *leu2-3*, *112 can1-100*) was a kind gift from Susan R. Wente’s lab (Ho *et al.* 1998). The strain SWY027 was cotransformed with plasmid pSW171 and plasmid encoding wt Snl1-L or Snl1-L mutants under galactose-inducible *GAL1* promoter. Cells were grown in SD growth medium supplemented with required amino acids at 23° for 3 d. Cells were adjusted to equal densities and serially diluted in 5-fold steps for spotting onto SD as well as SGal medium.

### Far-ultraviolet circular dichroism (CD) spectroscopy

CD spectra were recorded on a JASCO-J-815 spectropolarimeter. The spectra were recorded with a 1-mm pathlength cuvette with scan rate of 10 nm/min and averaged over three scans. The raw CD data were converted into Mean Residue ellipticity (Φ_MRE_), expressed as degrees square centimeter per decimole as follows:

$$\Phi_{\mathrm{MRE}}=\left(100^{*}\Phi_{\mathrm{obs}}\right)/\left[d^{*}C^{*}\left(n-1\right)\right]$$

Where Φ is the observed ellipticity (in degrees), d is path length (in centimeters), C is protein concentration (molar), and n is the total number of amino acids in the protein.

### Protein purification

The plasmid pPROEX-HTb-SNL1-S was transformed into *E. coli* strain Rosetta 2(DE3) (Invitrogen). The cells were grown at 37° until optical density at 600nm (OD) reaches 0.5−0.6. Culture was induced with 0.3 mM IPTG and further grown at 20° overnight. Cells were harvested and lysed in lysis buffer (25 mM HEPES, 150 mM NaCl, protease cocktail inhibitor) using lysozyme followed by sonication. Lysate was spun at 10,000 *g* for 45 min, and supernatant was loaded onto cobalt-based Talon metal affinity resin. Unbound protein was washed, and Snl1 was eluted with 300 mM imidazole and subsequently dialyzed using storage buffer (25 mM HEPES, 150 mM NaCl). Protein purity was verified using sodium dodecyl sulfate polyacrylamide gel electrophoresis and identity was confirmed using western blot analysis with anti-His tag antibody. Proteins were purified to near homogeneity (Supporting Information, Figure S1). If required to improve purity, protein was further purified by Superdex 75 gel-filteration chromatography.

### Western analysis

Cells grown in liquid culture was harvested using centrifugation and suspended in lysis buffer (phosphate-buffered saline with 0.2% Triton X-100 and protease inhibitor cocktail). Cells were lysed by vortexing with glass beads followed by sonication. Lysate was fractionated into supernatant and pellet by centrifugation at 10,000 *g* at 4°. Samples were boiled with loading dye for 15 min at 95°. Approximately 10 µg of total protein was loaded onto sodium dodecyl sulfate gels and transferred onto polyvinylidene fluoride membranes. Anti His-antibodies (Pierce Biotechnology) was used to capture His_6_-tagged Snl1.

## Results

### Deletion of Snl1 has no effect on [URE3] and [*PSI^+^*]

**Figure 1:**
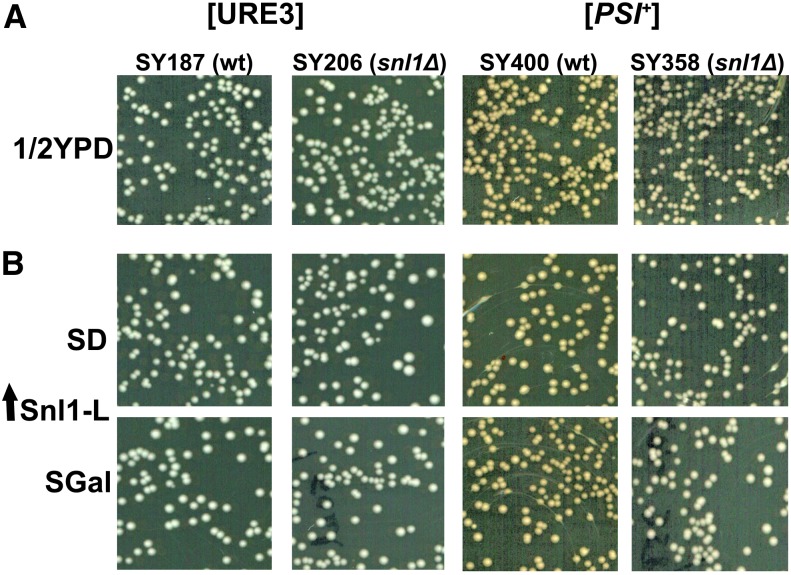
Snl1-L deletion or overexpression does not influence [URE3] or [*PSI^+^*] propagation. To monitor yeast prions, white colonies from 1/2 YPD plate were inoculated in liquid YPAD overnight, diluted to OD 0.02 in fresh YPAD, and further grown until OD 1.7. Cells were then spread onto 1/2YPD plates to obtain about 300−400 cells/plate and incubated at 30 for 3 d. Images show representative portions of the plates. (A) Effect of *SNL1* deletion on [URE3] and [*PSI^+^*] propagation. SY187 and SY206 are wild type (wt) and *SNL1* knockout strains, respectively, to monitor [URE3]. SY400 and SY358 are wt and *SNL1* knockout strains for monitoring [*PSI^+^*]. (B) Snl1-L was overexpressed from galactose-inducible promoter and transformants were used to monitor [URE3] and [*PSI^+^*] as described previously except that secondary cell cultures were grown in either noninducible dextrose (SD) or inducible galactose (SGal) medium. Uniformly white colony color indicates stable prion propagation.

Sse1, Fes1, and Snl1 are NEFs for cytosolic Hsp70s in *S. cerevisiae*. Snl1 is unique among these Hsp70 NEFs because it is localized on nuclear and ER membrane whereas the others are cytosolic. Sse1 and Fes1 are required for [URE3] propagation (Kryndushkin and Wickner 2007), as deletion of either *SSE1* or *FES1* results in loss of [URE3]. To examine the role of Snl1 in [URE3] propagation, *SNL1* was chromosomally deleted and [URE3] propagation monitored by colony color phenotype and ability to grow on solid growth medium lacking adenine (see *Materials and Methods*). As shown in Figure 1A, *snl1*Δ cells (strain SY206) propagated [URE3] like the wild-type (wt) strain (SY187), indicating that Snl1 was not essential for stable [URE3] propagation. We monitored [*PSI^+^*] similarly in isogenic strains SY358 and SY400. As for [URE3], disrupting *SNL1* had no effect on [*PSI^+^*] propagation. These results indicate that Snl1 is not essential for propagation of yeast [URE3] or [*PSI^+^*] prions.

### Overexpressed full-length Snl1 does not cure [URE3] and [*PSI^+^*]

The propagation of [URE3] and [*PSI^+^*] is sensitive to modulation of Ssa Hsp70 activity and thus influenced by changes in the abundance of Hsp70 co-chaperones such as Sse1 and Ydj1 (Moriyama *et al.* 2000; Fan *et al.* 2007; Kryndushkin and Wickner 2007; Sadlish *et al.* 2008; Sharma *et al.* 2009b). To test the effect of increased Snl1 expression on [URE3] and [*PSI^+^*] propagation, prions were monitored in strains transformed with plasmid encoding full length Snl1 (henceforth Snl1-L) under galactose inducible promoter. The pool of 6−7 transformants were further grown in minimal synthetic medium containing either dextrose (SD) or galactose (SGal) before spreading onto 1/2YPD plates. As shown in Figure 1B, cells pregrown in either SD or SGal showed uniform white colony color phenotype on 1/2YPD plate, indicating stable [URE3] propagation. These results suggest that [URE3] maintenance is not negatively influenced by increased Snl1 abundance although Snl1 encodes for a NEF for Hsp70. We also examined the effect of elevated Snl1-L on [*PSI^+^*] propagation in strain SY358 and SY400. Similar to as in case for [URE3], Snl1-L overexpression did not affect [*PSI^+^*] stability (Figure 1B).

**Figure 2:**
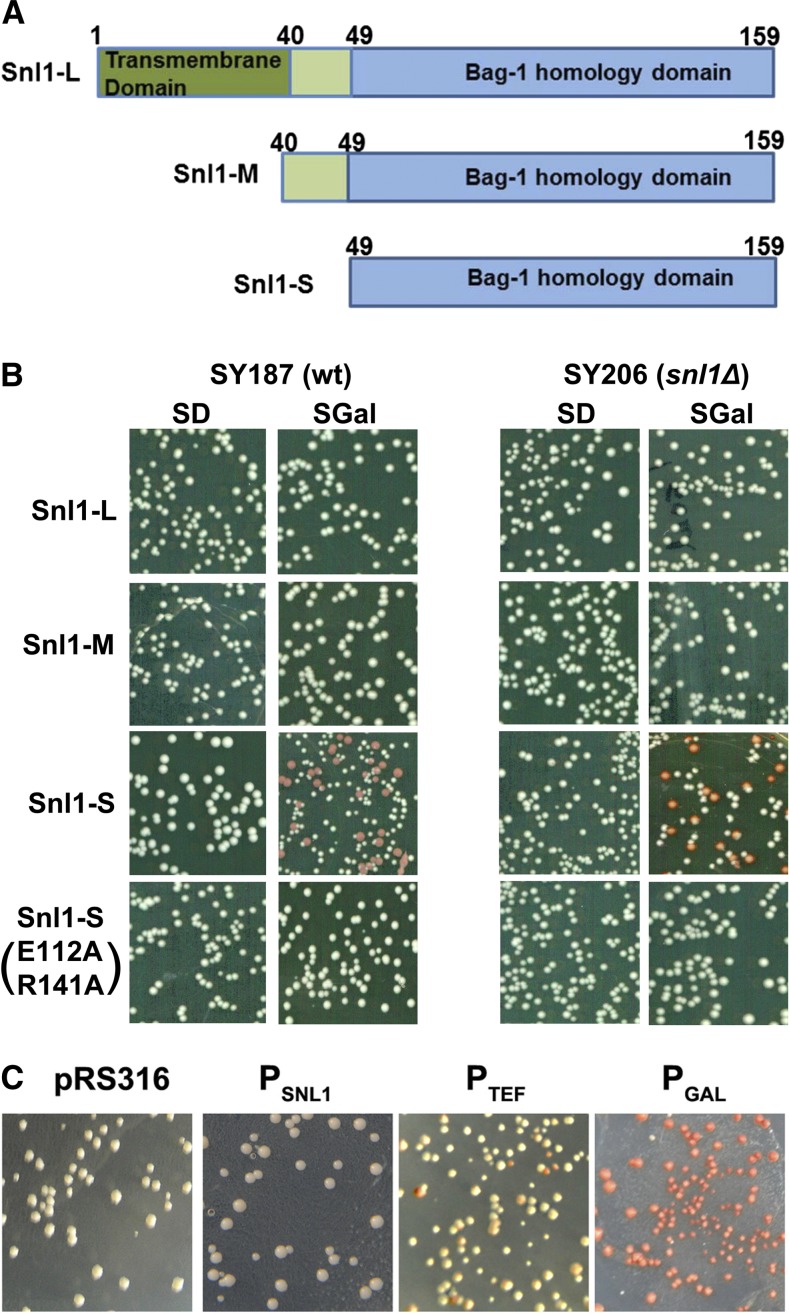
Snl1-S cures [URE3]. (A) Schematics of various Snl1 derivatives. (B) Cells carry plasmids for expressing intact and truncated Snl1 derivatives from galactose-inducible promoters. Overnight cultures were subcultured into medium containing 2% dextrose (SD) or 2% galactose (SGal). Shown are colonies on 1/2YPD plates after 3 d of incubation at 30° and 2 d at room temperature for color development. Colonies overexpressing Snl1-L or Snl1-M showed white phenotype, indicative of stable [URE3] propagation. Frequency of [ure-o] cells increased upon elevation of Snl1-S expression. Snl1-S(E112A,R141A) did not cure [URE3] as evident from white colony phenotype. (C) [URE3] was monitored in wild-type (wt) strain SY187 carrying plasmid pRS316 with or without Snl1-S controlled by its native Snl1 promoter (P_SNL1_), constitutive TEF promoter (P_TEF_), or galactose inducible promoter (P_GAL_).

Overproduction of Sse1, but not Fes1, significantly impairs [URE3] propagation, revealing a functional distinction among Hsp70 NEFs. The inability of Snl1-L overexpression to cure [URE3] could be because it is anchored on the nuclear and ER membrane or the manner by which it modulates Hsp70 activity. To test the former possibility, we expressed two previously reported Snl1 derivatives lacking the membrane spanning region (Figure 2A). One of these, Snl1(40−159) (henceforth Snl1-M), contains entire cytosolic domain and the second, Snl1(49−159) (henceforth Snl1-S), lacks the transmembrane domain as well as a nine-residue segment outside the region of Bag-1 domain homology. These truncated derivatives were similarly overexpressed from the *GAL1* promoter in both SY187 and SY206 strains and examined for their ability to cure [URE3] (Figure 2B). The frequency of [ure-o] colonies did not increase significantly upon overexpression of either Snl1-L or Snl1-M. In contrast, elevating Snl1-S in wt strain SY187 increased the frequency of [ure-o] cells by approximately 26 ± 0.3% ,as seen by increase in the number of red colonies on 1/2YPD plates when cells were pregrown in liquid medium containing galactose over that containing dextrose. To examine whether Snl1-L is involved in Snl1-S−dependent [URE3] curing, Snl1-S was similarly overexpressed in *snl1*Δ strain SY206. Snl1-S antagonized [URE3] in the *snl1*Δ strain, causing 42 ± 0.6% curing (Figure 2B).

Snl1-L is anchored to the membrane; however, Snl1-S is cytosolic. The increased [URE3] curing upon Snl1-S overexpression could be either attributable to an increase in Snl1-S level or merely the presence of Snl1-S in the cytosol. To distinguish between these possibilities, we expressed Snl1-S from its native Snl1 or stronger constitutive TEF promoter on a single-copy plasmid in wt strain SY187 (Figure 2C). The transformants were monitored for colony color phenotype as well as replicated onto plates lacking Ade. All transformants carrying empty plasmid or Snl1 under native Snl1 promoter showed white colony color phenotype when grown on limiting Ade medium (Figure 2C) and normal growth on medium lacking adenine (data not shown). Transformants carrying Snl1-S under relatively stronger *TEF* promoter showed approximately 5% of red color colonies with remaining colonies either red sectored (50%) or white. All transformants carrying Snl1-S under galactose-inducible promoter showed red colony color phenotype onto solid medium containing galactose. Together, these data show that the presence of Snl1-S in the cytosol is not enough to influence [URE3] propagation and thus its overexpression is required to destabilize [URE3]. Overall, these results suggest that elevated Snl1-S, but not Snl1-M or Snl1-L, antagonizes [URE3] propagation.

**Figure 3:**
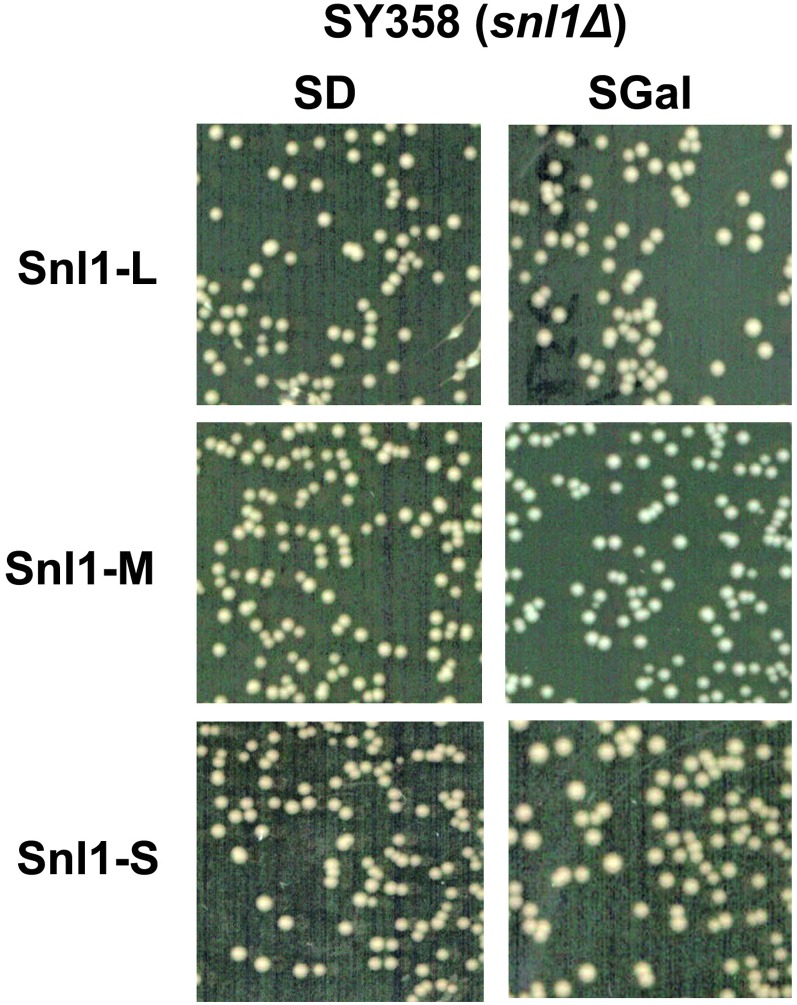
Overexpressed Snl1 or its derivatives does not antagonize [*PSI^+^*]. [*PSI^+^*] stability in cells overexpressing Snl1 or its truncated derivatives was monitored as described for [URE3]. The strain SY358 shows complete [*PSI^+^*] stability even upon overexpression of Snl1 or its derivatives. SD or SGal represents cells on 1/2YPD plate pregrown in liquid media containing 2% dextrose or 2% galactose, respectively.

We tested for effects of Snl1 and its derivatives on [*PSI^+^*] propagation by overexpressing them from a galactose inducible promoter in [*PSI^+^*] strains SY358 (Figure 3). As seen with [URE3], increasing abundance of Snl1-L and Snl1-M did not cure [*PSI^+^*]. Interestingly, although Snl1-S cured [URE3], it did not affect [*PSI^+^*] propagation.

### Interaction of Snl1-S with Hsp70 is important for [URE3] curing

The BAG1 domain of Snl1 interacts with Hsp70 *in vitro* and acts as its NEF. Residues E112 and R141 are indispensable for Hsp70 interaction and substitution of E112 or R141 to alanine abolishes Hsp70 interaction (Sondermann *et al.* 2002). To test whether Snl1-S−mediated curing of [URE3] requires Hsp70, the Snl1-S mutant having both E112 and R141 substituted by alanine, Snl1-S(E112A,R141A), was expressed from a galactose-inducible promoter in strains SY187 and SY206. As shown in Figure 2B, overproduced Snl1-S(E112A,R141A) did not affect [URE3] stability in either strain, indicating that Snl1-S requires interaction with Hsp70 to cure cells of [URE3].

**Figure 4:**
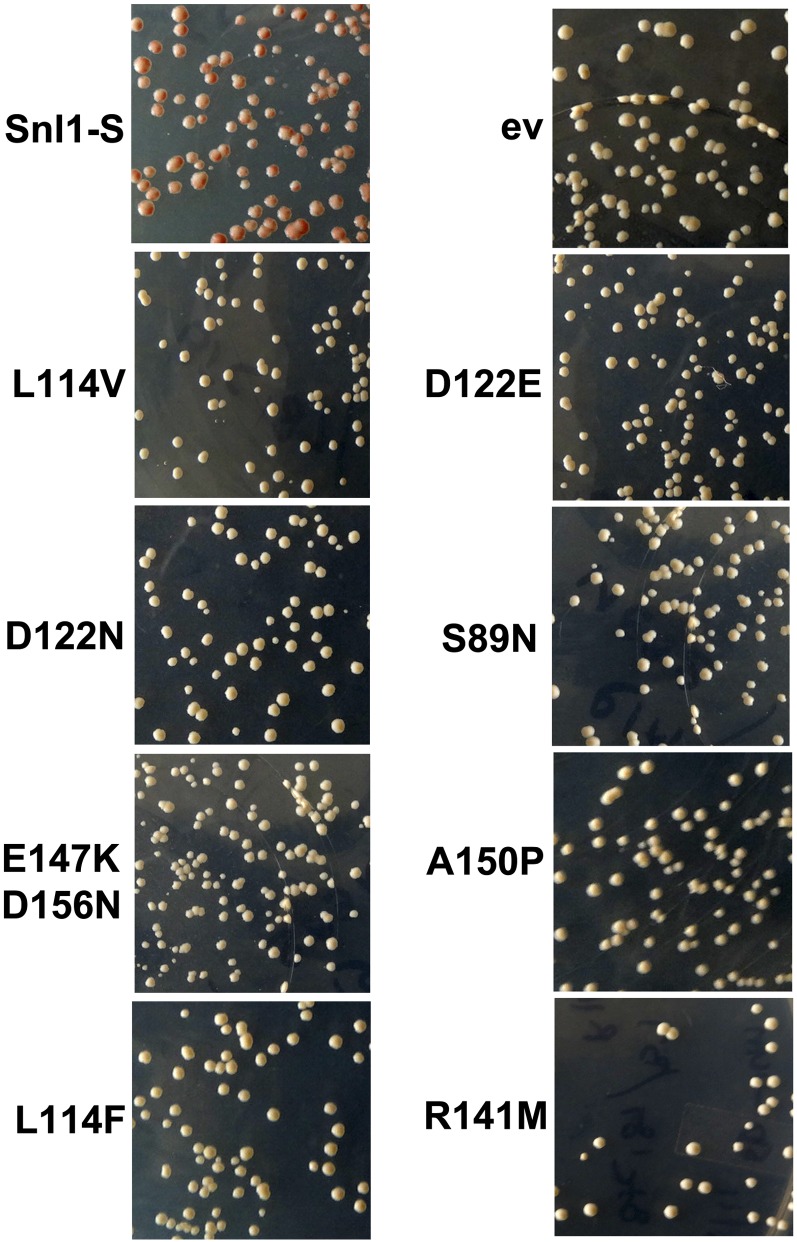
Snl1-S mutants unable to cure [URE3]. Plasmid pRS426-GPD-SNL1-S was randomly mutagenized using hydroxylamine as described in the *Materials and Methods* and subsequently transformed into wild-type strain SY187. Plasmid was extracted from white colonies and retransformed into SY187. The mutant identity of confirmed noncuring plasmids was determined by DNA sequencing. Shown are the [URE3] phenotypes upon overexpression of isolated Snl1-S mutants.

Human BAG domain proteins interact with multiple cellular factors in addition to Hsp70s. Although Snl1 interaction with cytsolic Hsp70s is long known, its interaction with other cellular partners is beginning to become appreciated. For example, it was shown recently that Snl1 associates with ribosomal proteins (Verghese and Morano 2012). To explore whether [URE3] curing by Snl1-S requires factors other than Ssa Hsp70, we randomly mutagenized Snl1-S to isolate mutants unable to antagonize [URE3]. When cells of strain SY187 were transformed by a high-copy plasmid (pRS426-GPD-SNL1-S) that express Snl1-S from the strong constitutive *GPD* promoter, more than 99.9% of resulting colonies were [ure-o] (Figure 4, top left panel). Plasmid pRS426-GPD-SNL1-S was mutagenized by hydroxylamine to generate a library of *SNL1* mutant alleles. The mutant library was transformed into strain SY187, and transformants were scored based upon the colony color phenotype, white being [URE3] and red as [ure-o]. Plasmids were extracted from [URE3] colonies and reconfirmed for their effect on [URE3] stability in strain SY187. As seen in Figure 4, several mutations spread over entire gene encoding Snl1-S, unable to cure [URE3], were identified.

We chose to focus on mutations that are not predicted to disrupt Hsp70 interaction. Snl1 structure is still unknown, but the crystal structure of BAG-1 domain with Hsc70 solved previously provides insight into BAG-1 domain residues important for Hsp70 interaction (Sondermann *et al.* 2001, 2002). Using the sequence alignment of Snl1-S with BAG-1 domain, we predicted homologous residues in Snl1 important for Hsp70 interaction. Table 3 describes the Snl1-S mutations obtained from random screen, the homologous residues in the human BAG-1 domain, and the predicted Hsc70 interacting residue with human BAG-1. As expected various mutants (D122N, E147KD156N, R141M) with substitutions at positions crucial for Hsp70 interaction were obtained from the screen, suggesting that our random mutagenesis approach can successfully screen potential Snl1 mutants unable to cure [URE3]. Among various mutants, L114F, L114V, S89N, and A150P are not predicted to affect Hsp70 interaction. Because proline is known to disrupt secondary structural elements such as α-helix and β-sheet, and Leu at position 114 is an aliphatic residue, we chose Snl1-S(L114V) and Snl1-S(S89N) to examine the underlying basis of their detrimental effect on ability of Snl1-S to cure [URE3].

**Table 3:** Snl1 mutants obtained from random mutagenesis that is unable to cure [URE3]

Snl1 Mutation	BAG-1 Residue	Hsc70 Interaction
S89N	Q187	Not known
D122N	D222	R262
E147K	K243	Not known
D156N	D252	R258
R141M	R237	Y294, E283
L114F	F214	Not known
L114V	F214	Not known
A150P	A246	Not known

Corresponding wild-type residues in BAG-1 protein and its Hsc70-interacting residues also are shown.

**Figure 5:**
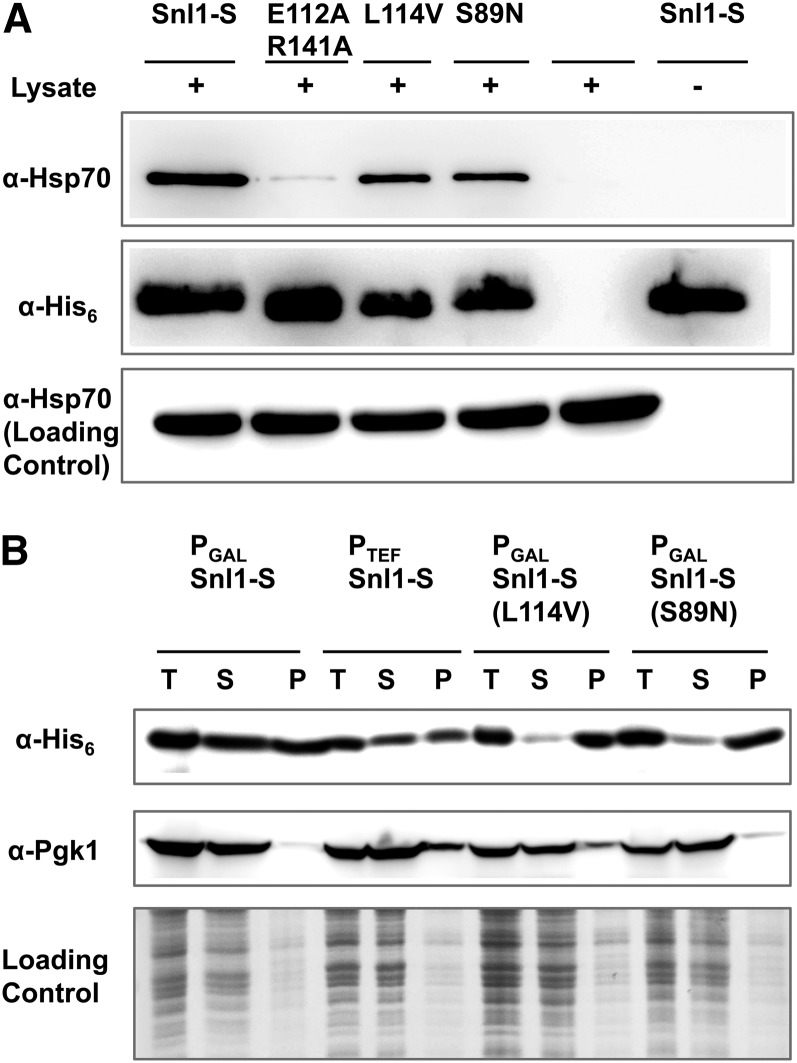
Snl1-S(S89N) and Snl1-S(L114V) interact with Hsp70. (A) His_6_-tagged wild-type Snl1-S or its mutants were adsorbed onto metal affinity resin and further incubated with yeast lysate from wild-type strain SY187. The bound fraction from yeast lysate was analyzed for Hsp70 on immunoblot using anti-Hsp70 antibodies. As seen, Hsp70 was detected from beads incubated with either wild-type Snl1-S, Snl1-S(L114V) or Snl1-S(S89N), but not as much from beads incubated with the Snl1-S(E112A,R141A). The middle panel shows the relative amounts of Snl1-S (wt or mutants) bound on the resin. Total yeast lysate incubated with resin was probed using with anti-Hsp70 antibodies to confirm equal load of Hsp70 (lower panel). (B) Upper panel shows the relative abundance of Snl1-S(L114V) or Snl1-S(S89N) compared with wild-type Snl1-S on immunoblot. Snl1-S(L114V) or Snl1-S(S89N) was expressed from galactose inducible promoter. Snl1-S(wt) was expressed either from galactose-inducible or *TEF* promoter. The whole yeast lysate (T) was fractionated into soluble (S) and pellet (P) fractions and blots probed using antibodies against the Hexa-his tag. Middle panel shows immunoblot using anti-Pgk1 antibody as loading and fractionation control. Lower panel shows Coomassie Brilliant Blue staining of same samples as loading control.

To determine whether these mutations affect Hsp70 binding, the mutant proteins were purified and examined for their ability to interact with Hsp70 *in vitro*. As described in the *Materials and Methods*, a hexa-Histidine tag (His_6_-tag) was added to the N-terminus of wt Snl1-S and the mutant proteins. The purified proteins were bound to cobalt-based metal affinity resin and then incubated with yeast cell lysate from wt strain SY187. The unbound fraction was washed and bound protein examined by immunoblot via the use of anti-Hsp70 antibodies. As expected, Hsp70 coprecipitated with wt Snl1-S and not using Snl1-S(E112A,R141A) (Figure 5A). However, binding of Snl1-S(L114V) or Snl1-S(S89N) to Hsp70 was similar to that of Snl1-S. Thus, Snl1-S(L114V) and Snl1-S(S89N) interacted normally with Hsp70, which implies something in addition to Hsp70 interaction is necessary for Snl1-S to cure [URE3].

To examine whether the inability of Snl1-S(L114V) and Snl1-S(S89N) to cure [URE3] was attributable to alteration in their cellular abundance, the C-terminally His_6_-tagged mutant proteins were expressed from galactose-inducible promoter in SY187 and the protein level was compared with that of wt Snl1-S expressed from either galactose inducible promoter or TEF promoter. The His_6_-tagged wt Snl1-S cured [URE3] to an extent similar to that of untagged Snl1-S (Figure S2), indicating that the C-terminal His_6_-tag does not affect Snl1-S curing activity. The cell lysate from [ure-o] cells was fractionated into soluble and pellet fractions and the protein level assessed by probing immunoblots with anti-His_6_ antibodies (Figure 5B). We found similar abundance of Snl1-S, Snl1-S(L114V), and Snl1-S(S89N) proteins in whole cell lysate. The wt Snl1-S fractionated into both soluble and insoluble fraction. The presence of wt Snl1-S into insoluble fraction suggests that either the N-terminal membrane anchoring region or membrane association is important to maintain overall stability of the protein structure. Snl1-S was fractionated more in the soluble fraction as compared to Snl1-S(L114V) and Snl1-S(S89N), suggesting that the inability of the mutant proteins to cure [URE3] could be due to its decreased solubility.

**Figure 6:**
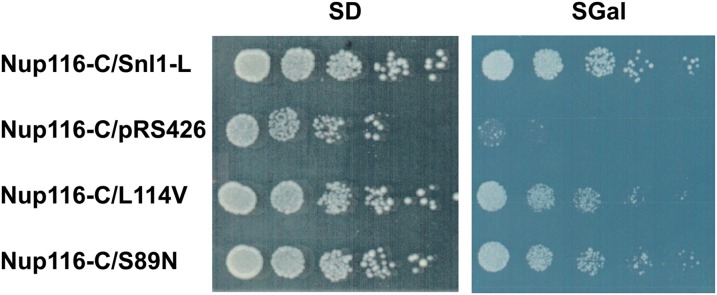
Snl1-L(S89N) and Snl1-L(L114V) rescue Nup116-C−mediated toxicity. Strain SWY027 (*nup116Δ*) was cotransformed with pSW171 (to express Nup116-C) and either wild-type Snl1-L, empty plasmid, Snl1-L(L114V), or Snl1-L(S89N). Cells were grown at 23° and then serially diluted and spotted onto medium containing 2% dextrose (SD) or 2% galactose (SGal). On galactose-containing medium, similar growth is seen for wild-type -Snl1-L, Snl1-L(S89N), or Snl1-L(L114V), indicating that both Snl1-L(S89N) and Snl1-L(L114V) provide Snl1 function required to protect SWY027 from Nup116-C toxicity.

Overexpression of Nup116-C from a galactose-inducible promoter arrests cell growth in *nup116Δ* strain, an effect that is suppressed by overexpressing Snl1-L (Ho *et al.* 1998; Sondermann *et al.* 2002). The rescue of cell growth by Snl1-L requires Hsp70 as mutations that inhibit Snl1-L ability to interact with Hsp70 are unable to suppress *nup116Δ* phenotype. We examined whether Snl1-L(L114V) and Snl1-L(S89N) also suppress *nup116Δ* phenotype. Interestingly, both Snl1-L(L114V) and Snl1-L(S89N), which were unable to cure [URE3], suppressed Nup116-C−mediated cellular toxicity (Figure 6). The rescue of Nup116-C toxicity by these mutants indicates that the substitutions do not significantly affect activity or abundance of the full-length Snl1-L required to suppress the toxicity.

### Curing of [URE3] by Snl1-S is Hsp70 isoform independent

Like most eukaryotes, *S. cerevisiae* possesses multiple cytosolic Hsp70 isoforms, designated Ssa1-4 in yeast. We showed previously that though highly homologous, the constitutively expressed Ssa Hsp70 isoforms, Ssa1 and Ssa2 function distinctly with regard to yeast prion propagation and vacoular mediated degradation (Sharma and Masison 2011). The functional distinction between Ssa1 and Ssa2 is regulated by the ATPase domain of Hsp70. Because BAG domain proteins are known to interact with Hsp70 ATPase domain, we monitored [URE3] loss upon overproduction of Snl1-S in strains expressing either Ssa1 (A1) or Ssa2 (A2) as sole source of cytosolic Ssa Hsp70.

**Figure 7:**
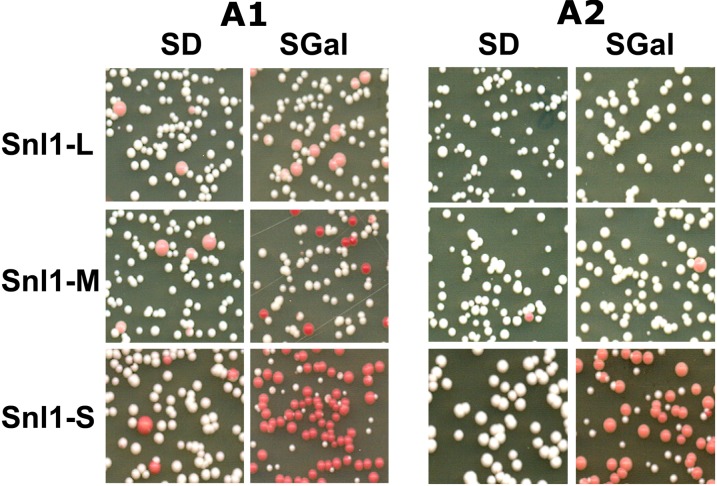
Snl1-S cures [URE3] in Ssa1 and Ssa2 strain with similar efficiency. [URE3] curing was monitored as described in Figure 2. Cells were grown in SD or SGal medium and spread onto 1/2YPD plates and incubated 3 d at 30° and 2 d at room temperature. Strains are expressing either Ssa1 (A1) or Ssa2 (A2) as sole source of Ssa Hsp70. [URE3] is unstable in strain A1 as seen from cells grown in SD. Overexpression of Snl1-S, but not other Snl1 derivatives, increases [URE3] curing significantly as seen by increase in number of red colonies.

We previously reported that [URE3] is unstable in strain A1 and thus approximately 20% of A1 cells lose [URE3] (Sharma *et al.* 2009a) when grown in rich medium containing adenine. In contrast, [URE3] is stable in A2 cells, and its loss, if any, is very infrequent. To examine the effect of Snl1-S overexpression, A1 and A2 cells harboring galactose-inducible *SNL1-S* were grown overnight in dextrose medium lacking adenine to select for [URE3] cells. The cultures were then split into SD or SGal medium and grown from OD_600nm_ of 0.02−1.7 (roughly six to seven generations). Cells were then plated onto 1/2YPD plates for monitoring [URE3]. Under these conditions, 5 ± 1% of A1 cells lost [URE3] even in noninducing medium (Figure 7). The overproduction of Snl1-S in strain A1 resulted in increase of [ure-0] cells to 61 ± 9.9% of [ure-o] cells. Similarly, [ure-o] cells in strain A2 cultures increased to about 51 ± 0.5% upon Snl1-S overproduction. These data suggest that the curing is Hsp70 isoform independent. As seen for wt strain SY187, as compared to Snl1-S, Snl1-L and Snl1-M did not cure [URE3] significantly.

### Snl1 derivatives have differences in secondary structural content

**Figure 8:**
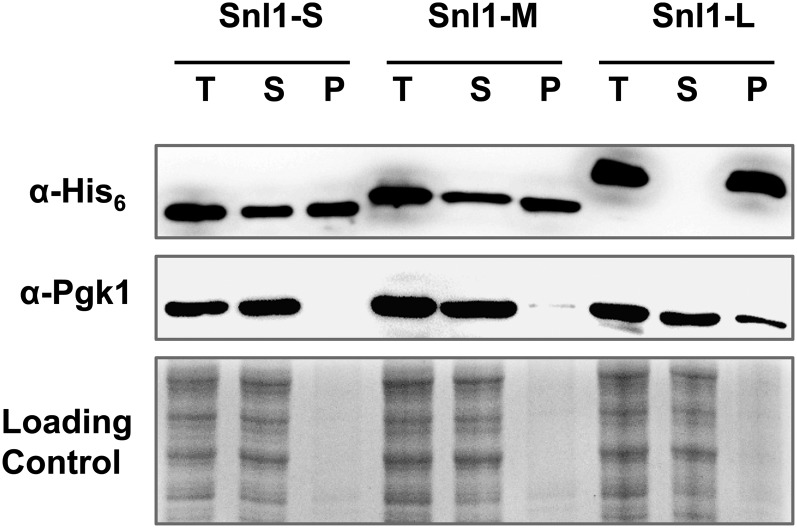
Expression level and solubility of Snl1 derivatives. Western analysis of samples (10 µg) of whole cell lysate was performed with anti-His_6_ antibody. Yeast whole lysate (T) was centrifuged at 10000 g to fractionate into soluble (S) and pellet (P) fractions. The middle panel shows immunoblot using anti-Pgk1 antibody as loading and fractionation control. As expected, Pgk1 was detected primarily in whole cell lysate or supernatant. The lower panel shows Coomassie Brilliant Blue staining of same samples as loading control.

Snl1-M and Snl1-S interact with Hsc70 with similar binding affinity (*K*_D_ = 5 µM), act as NEFs of Hsc70, and stimulate dissociation of Hsp70-substrate complex with similar efficiency (Sondermann *et al.* 2002). Both derivatives also show similar association with ribosomes (Verghese and Morano 2012). However, overexpression of only Snl1-S and not Snl1-M results in [URE3] loss. To examine whether the difference in [URE3] curing is attributable to differences in their cellular abundance, the relative abundance of hexa-His-tagged versions of all Snl1 derivatives was monitored. Cells harboring Snl1 or its derivatives were induced in galactose medium, and protein expression was monitored in whole-cell lysate, supernatant, and pellet. As evident from Figure 8, all proteins were expressed at similar levels. As expected, because of its membrane association, Snl1-L was primarily present in the pellet. The cytosolic Snl1 derivatives, Snl1-M and Snl1-S, partitioned similarly in supernatant and pellet fractions. These results suggest that the distinct effects of Snl1-M and Snl1-S were not caused by a difference in their cellular abundance.

**Figure 9:**
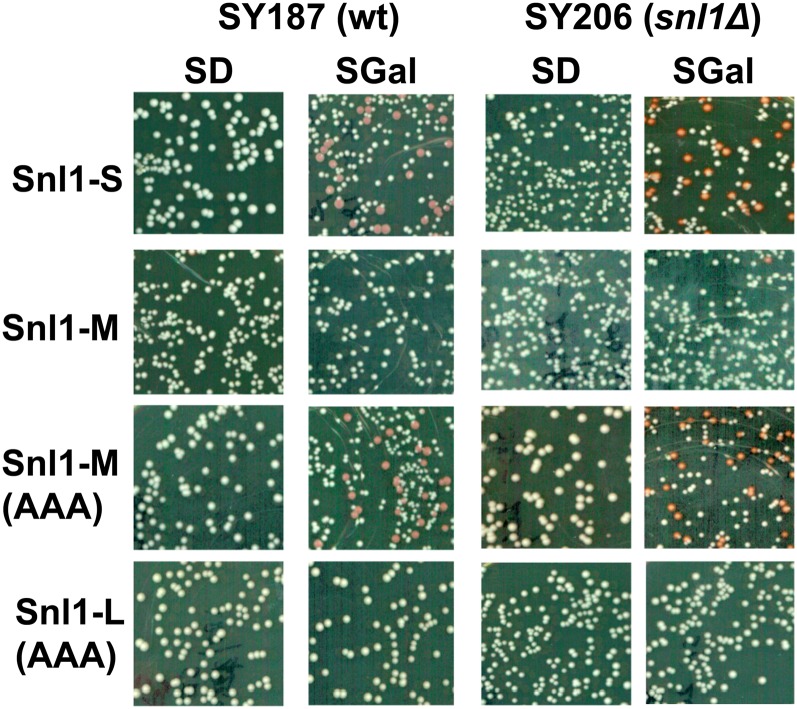
Snl1-M(AAA) mutant cures [URE3]. [URE3] curing was monitored as described in Figure 2. Indicated Snl1 derivatives were overexpressed from galactose-inducible promoter and [URE3] curing monitored in wild type (wt; SY187) and *snl1Δ* (SY206) strains. Increase in [URE3] curing was seen only in Snl1-S and Snl1-M(AAA) mutant.

Snl1-M differs from Snl1-S only by the presence of a nine-amino acid segment (GSKGKSSKK) at its N-terminus, which has a profound effect on [URE3] curing ability. This segment is rich in Ser and Lys residues, but their significance for *in-vivo* function is not clear. To explore the significance of these amino acids, lysines at position 42, 44, and 47 were simultaneously substituted by alanine to produce Snl1-M(AAA), and the mutant protein was overexpressed from galactose-inducible promoter as described previously (Figure 9). Interestingly, Snl1-M(AAA) cured [URE3] as efficiently as Snl1-S, clearly showing that these N-terminal lysine residues act to inhibit Snl1-M from curing [URE3]. To examine whether similar residues effect [URE3] curing by Snl1-L, the same residues were mutated to alanine to generate construct Snl1-L(AAA). The mutation of the lysine residues in full-length Snl1-L had no effect on its ability to cure [URE3], suggesting that something other than these residues, such as membrane association, contributed to the inability of Snl1-L to cure [URE3].

**Figure 10:**
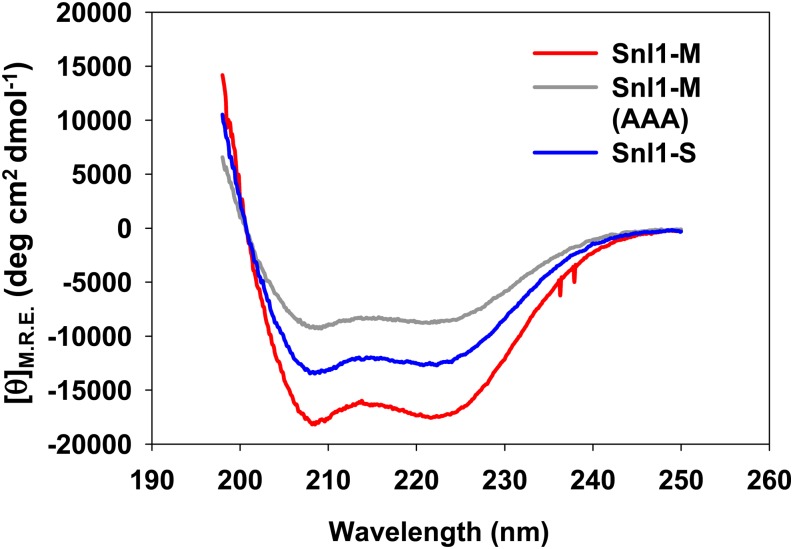
Far-ultraviolet circular dichroism spectroscopic analysis of Snl1 and its derivatives. A total of 10 µM protein in buffer 2.5 mM HEPES, 15 mM NaCl, pH 7.5 in a 1-mm path length cuvette was used to record CD spectra. In each case average of three spectra is reported. As seen Snl1-S has less helical content than Snl1-M. Mutation of three lysine residues to Ala further reduces secondary structural content of the protein.

To examine the structural properties of Snl1-M and Snl1-S, these derivatives were expressed in *E. coli* and purified with Talon cobalt affinity resin. The secondary structural content of Snl1-M and Snl1-S was examined using far-ultraviolet CD between 195 and 250 nm, which provides characteristic spectra for different structural elements such as α-helix, β-sheet or random coil. As shown in Figure 10, the CD spectrum of both Snl1-M and Snl1-S showed a double minimum at 222 nm and 208 nm and a high peak at 198 nm, which is characteristic of α-helical proteins. The negative Cotton effect at 222 nm and 208 nm is greater for Snl1-M compared with that obtained from Snl1-S, indicating although both are primarily α-helical, Snl1-S has lower α-helical content than Snl1-M. To examine whether there is any correlation between the secondary structure of the protein and its ability to influence [URE3], we also measured the CD spectrum of Snl1-M(AAA) mutant. Interestingly, the mutant protein showed lower α-helical content than the parent wt protein as well as Snl1-S suggesting, that increased flexibility of Snl1-S structure could be the basis of functional differences between Snl1-S and Snl1-M.

## Discussion

The only BAG domain protein identified in *S. cerevisiae* is Snl1, which is known to be anchored on the ER and nuclear membrane. Similar to other BAG domain proteins, the BAG domain of Snl1 stimulates ATPase activity of Hsp70s in the presence of Hsp40 proteins. Because Snl1 interacts with Ssa Hsp70, it is imagined that Snl1 could influence the refolding of aggregation-prone cytosolic Hsp70 substrates. In the present study we show that overexpressing the Bag1 homology domain of Snl1, the least studied of the three yeast cytosolic Hsp70 NEFs, cured cells of [URE3]. However, deleting Snl1 did not affect propagation of [*PSI^+^*] and [URE3] prions. In contrast, deleting either of the other NEFs, Sse1 or Fes1, inhibits propagation of both [*PSI^+^*] and [URE3] prions (Fan *et al.* 2007; Kryndushkin and Wickner 2007). Together, these data suggest that Sse1 and Fes1 possess activities important for normal propagation of prions in yeast and that Snl1 lacks this activity.

Overexpressing intact Snl1 (Snl1-L) was like Fes1 in that it did not affect either [*PSI^+^*] or [URE3]. However, overexpressing just the Bag1 homology region of Snl1 (Snl1-S) was like Sse1 in that it cured cells of [URE3], but not [*PSI^+^*]. These findings suggest that the normal tethering of Snl1 to ER and nuclear membrane restricts its ability to affect prions. This conclusion is supported by our findings that reducing the lysine content of the amino-terminal tail of Snl1-M allowed it to cure cells of [URE3], but the same modification of the full-length Snl1-L containing the transmembrane region did not. These observations raise the notion that Fes1 might have an anti-[URE3] activity that is normally dampened by some other aspect of Fes1 function.

Like Sse1, the ability of Snl1 to inhibit prion propagation depends on its ability to interact with Hsp70. However, the S89N and L114V mutation, which prevented Snl1-S from curing [URE3], did not disrupt the known activities of SnL1 to interact with Hsp70 or to perform its role related to the nuclear pore function of Nup116. Therefore, these residues are critical for a previously unrecognized function of the Snl1 Bag-1 domain that is different from the known roles of Snl1 in the cell. Because of the importance of Hsp70 and other components of the chaperone machinery for propagation of yeast prions, we suspect that this activity of Snl1 is related to how it cooperates with or regulates this machinery. It is also possible that the membrane association of Snl1-L(S89N) and Snl1-L(L114V) prevents their ability to form inclusions *in vivo*. This hypothesis is also supported by the fact that Snl1-S, which lacks membrane association, also forms insoluble inclusion bodies.

Interestingly the elevated Snl1-M, containing entire cytosolic Snl1 domain, that consist of nine additional residues at N-terminus of Snl1-S is unable to cure [URE3]. Because both Snl1-M and Snl1-S show similar affinity for Hsc70 *in vitro*, it is unlikely that the N-terminal extension has any role in inhibiting Hsp70 interaction. CD shows that Snl1-M has greater helical content than Snl1-S, which could be attributable to the presence of larger loops or other flexible region in Snl1-S that decreases secondary structural content. Such flexible regions in the protein increase dynamics of protein motion, resulting in increased adaptability to changes in protein structure upon intermolecular interaction. In agreement with this, the Snl1-M(AAA) that cures [URE3] also shows lower helical content than wt Snl1-M. Alternatively, it is also possible that the N-terminal extension either interacts with some other cellular factor or the Hsp70 substrate directly that in turn interferes with [URE3] curing. The interaction region of BAG-2 domain with Hsp70 client proteins is known to overlap with Hsp70 binding site (Xu *et al.* 2008). Similar interaction if present for Snl1-M might interfere with Hsp70 ability to remodel prion aggregates. The substitution of lysine residues to alanine in Snl1-M restores its ability to cure [URE3] indicating specific role of these residues in the prion propagation. In a recent report, Verghese and Morano (2012) show that the lysine-rich region from amino acids 52−58 in Snl1 mediates its interaction with ribosomes. Because the deletion of first 50 amino acids has no effect on its ribosomal association, the substitution of lysines to alanine at position 42, 44, and 47 should not affect the ability of Snl1-M to interact with ribosomes. These observations suggest that inability of Snl-M to cure [URE3] could be independent of its ability to interact with ribosomes. Together, our mutational analysis of Snl1 as well as results from Snl1 derivatives reveal that though Hsp70 interaction is critical for [URE3] propagation, something in addition to this activity seems to be necessary for Snl1 to inhibit propagation of [URE3].

The *SNL1* knockout strain does not show any detectable phenotype at 30° and thus the exact *in vivo* significance of Snl1 is still not clear. In humans, three BAG-1 isoforms are expressed as the result of alternative in-frame translational start sites, and the three isoforms perform overlapping as well as distinct cellular functions. The functional diversity of BAG-1 is contributed by various domains constituting BAG domain protein. Previous attempts to understand the functional significance of various regions of Snl1 show no significant differences between the BAG homology domain and entire cytosolic region of the protein. One such study has revealed a small stretch of amino acids within BAG homology domain that mediates its association with ribosomal proteins (Verghese and Morano 2012). The present study extends our understanding about the significance of N-terminal region at the BAG domain of Snl1 and suggests that this region might act as a regulatory motif of at least some of its *in vivo* functions.

## Supplementary Material

Supporting Information
