# Supplementary material for: The BAG Homology Domain of Snl1 Cures Yeast Prion [URE3] Through Regulation of Hsp70 Chaperones
Source: G3 (Bethesda). 2014 Mar 13;4(3):461–70. doi: 10.1534/g3.113.009993 (PMC3962485; doi:10.1534/g3.113.009993)
Supplement: Supporting Information [file supp_4_3_461__index.html]

The BAG Homology Domain of Snl1 Cures Yeast Prion [URE3] Through Regulation of Hsp70 Chaperones — Supporting Information 

# The BAG Homology Domain of Snl1 Cures Yeast Prion [URE3] Through Regulation of Hsp70 Chaperones

## Supporting Information for Kumar *et al.*, 2014

**Files in this Data Supplement:**

- Supporting Information - Figures S1-S2 (PDF, 443 KB)
- Figure S1 - Coomassie brilliant blue staining of purified Snl1 derivatives after elution from Talon metal affinity resin. (PDF, 321 KB)
- Figure S2 - The presence of C-terminal His6-tag does not affect Snl1-S ability to antagonize [URE3]. (PDF, 384 KB)
