# Supplementary material for: The BAG Homology Domain of Snl1 Cures Yeast Prion [URE3] Through Regulation of Hsp70 Chaperones
Source: G3 (Bethesda). 2014 Mar 13;4(3):461–70. doi: 10.1534/g3.113.009993 (PMC3962485; doi:10.1534/g3.113.009993)
Supplement: Supporting Information [file supp_g3.113.009993_009993SI.pdf]

G3 Genes | Genomes | Genetics

The BAG Homology Domain of Snl1 Cures Yeast Prion [URE3] Through Regulation of Hsp70 Chaperones

Navinder Kumar[§], Deepika Gaur[§], Daniel C. Masison[†] and Deepak Sharma[§]*
[§]Council of Scientific and Industrial Research-Institute of Microbial Technology, India and [†]Laboratory of Biochemistry and Genetics, National Institutes of Diabetes and Digestive and Kidney Diseases, USA.

*Corresponding author: E-mail: deepaks@imtech.res.in, Institute of Microbial Technology, Sector 39A, Chandigarh, India. Phone : 91-172-6665478

DOI: 10.1534/g3.113.009993

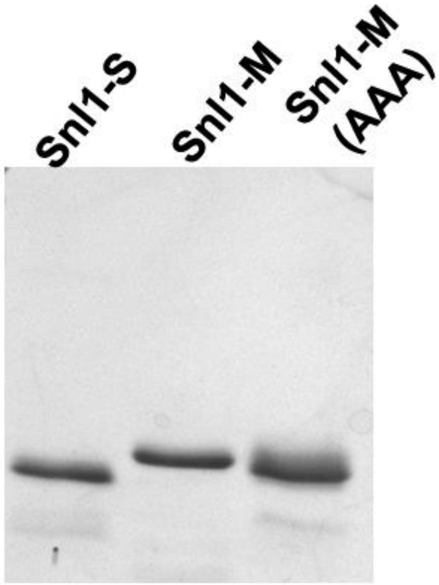

Figure S1 Coomassie brilliant blue staining of purified Snl1 derivatives after elution from Talon metal affinity resin. The minor impurity seen could be the degradation product of Snl1 derivatives as it also appears upon protein storage at 4°C.

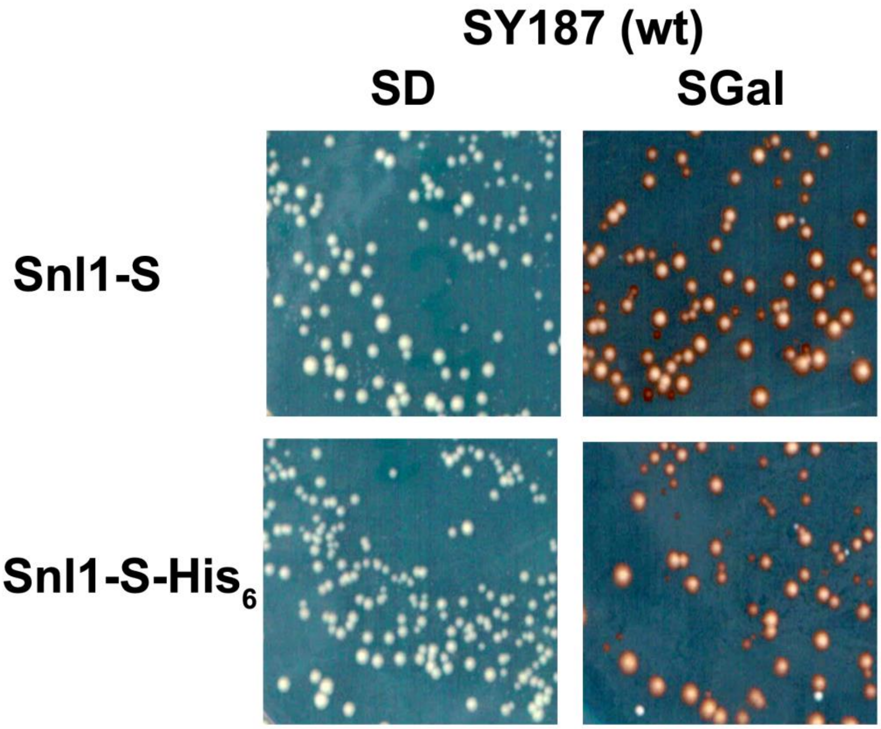

Figure S2 The presence of C-terminal His$_6$-tag does not affect Snl1-S ability to antagonize [URE3]. The strain SY187 was transformed with plasmid encoding Snl1-S or C-terminal His$_6$ tagged Snl1-S under galactose inducible promoter. Transformants were spread onto plate containing either dextrose or galactose containing minimal growth media lacking uracil for plasmid selection and limiting adenine to monitor [URE3]. As seen both constructs antagonized [URE3] with similar efficiency.
