# Supplementary material for: The BAG Homology Domain of Snl1 Cures Yeast Prion [URE3] Through Regulation of Hsp70 Chaperones
Source: G3 (Bethesda). 2014 Mar 13;4(3):461–70. doi: 10.1534/g3.113.009993 (PMC3962485; doi:10.1534/g3.113.009993)
Supplement: Supporting Information [file supp_g3.113.009993_FigureS1.pdf]

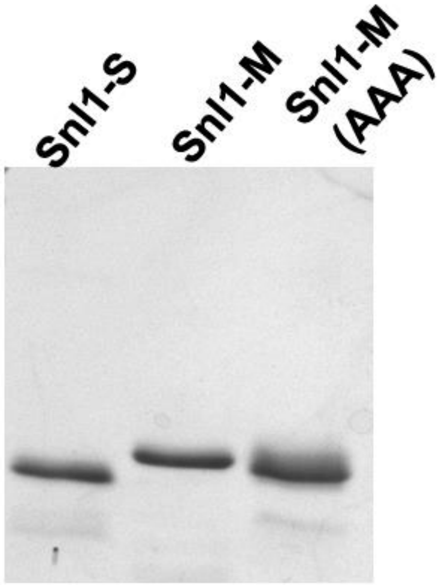

Figure S1 Coomassie brilliant blue staining of purified Snl1 derivatives after elution from Talon metal affinity resin. The minor impurity seen could be the degradation product of Snl1 derivatives as it also appears upon protein storage at 4°C.
