# Supplementary material for: The BAG Homology Domain of Snl1 Cures Yeast Prion [URE3] Through Regulation of Hsp70 Chaperones
Source: G3 (Bethesda). 2014 Mar 13;4(3):461–70. doi: 10.1534/g3.113.009993 (PMC3962485; doi:10.1534/g3.113.009993)
Supplement: Supporting Information [file supp_g3.113.009993_FigureS2.pdf]

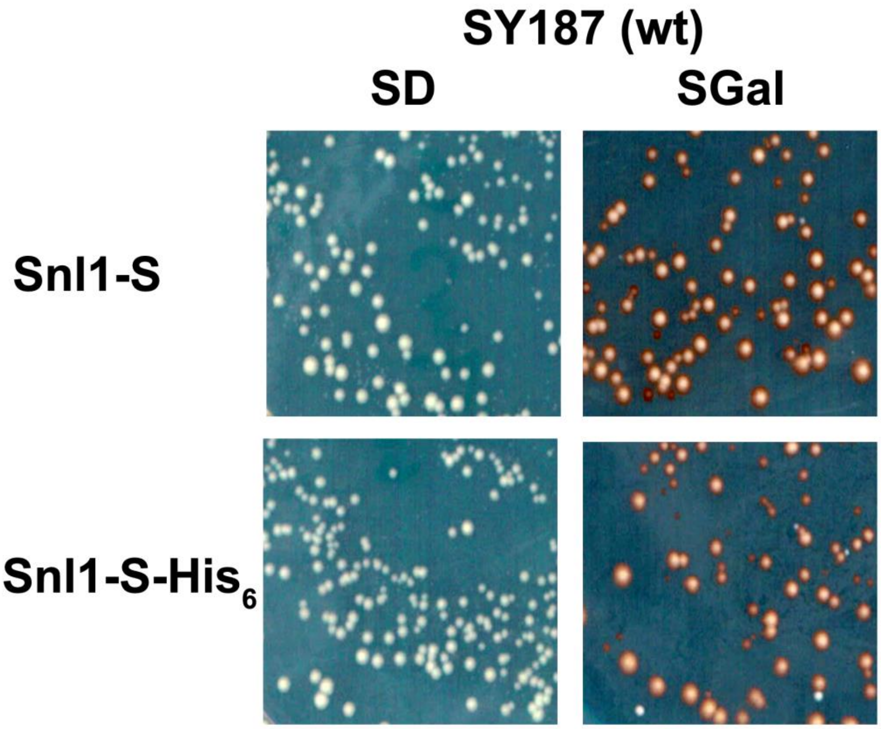

Figure S2 The presence of C-terminal His_6-tag does not affect Snl1-S ability to antagonize [URE3]. The strain SY187 was transformed with plasmid encoding Snl1-S or C-terminal His_6 tagged Snl1-S under galactose inducible promoter. Transformants were spread onto plate containing either dextrose or galactose containing minimal growth media lacking uracil for plasmid selection and limiting adenine to monitor [URE3]. As seen both constructs antagonized [URE3] with similar efficiency.
